# Structure, Dynamics, and Accurate Laboratory Rotational Frequencies of the Acrylonitrile–Methanol Complex

**DOI:** 10.1021/acs.jpca.0c01334

**Published:** 2020-04-11

**Authors:** Camilla Calabrese, Assimo Maris, Annalisa Vigorito, Sergio Mariotti, Pantea Fathi, Wolf D. Geppert, Sonia Melandri

**Affiliations:** † Dipartimento di Chimica “G. Ciamician”, Università di Bologna, via Selmi 2, I-40126 Bologna, Italy; ‡ Departamento Química Física, Facultad de Ciencia y Tecnología Universidad del País Vasco (UPV/EHU), Apartado 644, E-48080 Bilbao, Spain; § Biofisika Institute, (CSIC, UPV/EHU), University of the Basque Country (UPV/EHU), Barrio Sarriena, S/N, 48940 Leioa, Spain; ∥ INAF - Osservatorio di Radioastronomia, via P. Gobetti, 101, I-40129 Bologna, Italy; ⊥ Department of Physics, Stockholm University, Albanova University Center, SE-106 91 Stockholm, Sweden

## Abstract

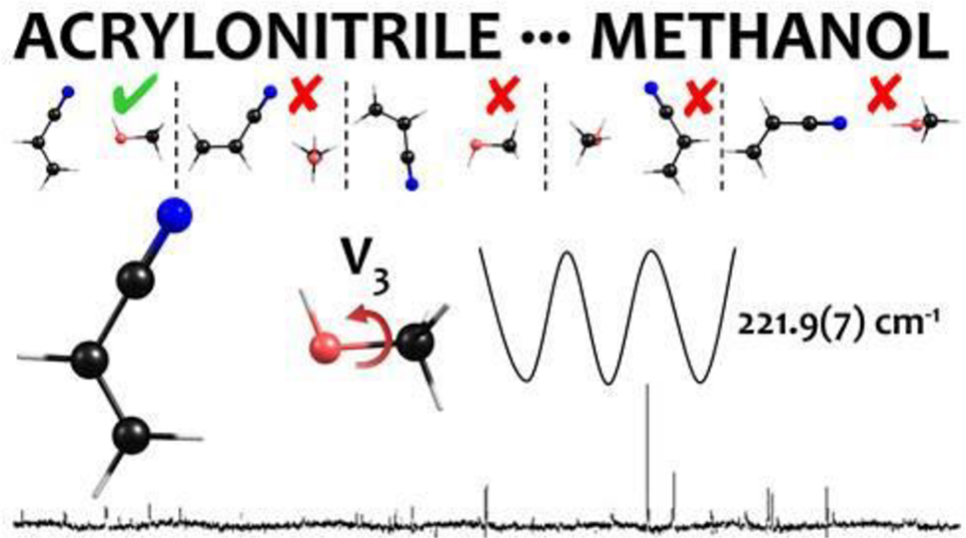

The hydrogen-bonded complex between acrylonitrile (CH_2_=CHCN) and methanol has been characterized spectroscopically in the millimeter wave range (59.6–74.4 GHz) using a free jet absorption millimeter wave spectrometer. Precise values of the rotational and centrifugal distortion constants were obtained from the measured frequencies of the complex of acrylonitrile with CH_3_OH and CD_3_OD. The analysis of the splittings of the rotational lines due to the hindered internal rotation of the methanol methyl group led to the determination of a *V*_3_ value of 221.9(7) and 218(5) cm^–1^ for the complexes of CH_3_OH and CD_3_OD, respectively, and these values are about 40% lower than that of free methanol. The structure of the observed conformation is in agreement with the global minimum determined at the MP2/aug-cc-pVTZ level of calculation, and the counterpoise corrected intermolecular binding energy, obtained at the same theoretical level, is *D*_e_ = 26.3 kJ mol^–1^.

## Introduction

Hydrogen-bonded clusters have been in the constant focus of interest of theoretical and experimental chemists. Despite the noncovalent character of the hydrogen bond (HB), they can be quite stable, and hydrogen-bonded species (especially water clusters) have been found in a multitude of environments including planetary atmospheres. In the terrestrial atmosphere, hydrogen-bonded water clusters can act as templates for formation of larger entities. This way, large water clusters of both cations and anions can be formed, which appear downward from the D-layer of the terrestrial ionosphere (60–100 km altitude). Earlier rocket-based missions detected a predominance of H_3_O^+^(H_2_O)_*n*_ clusters between altitudes of 35 and 53 km.^1−3^ Such species can act as nucleation sites for the formation of aerosols, which greatly affect planetary climates including our own.

Some classes of molecules (e.g., alcohols, acids, amides) are very prone to HB formation, acting either as a proton donor or acceptor. The electron pair located at the nitrogen renders nitriles (or cyano compounds) quite efficient proton acceptors that can easily undergo hydrogen bonding with proton donors like water, alcohols, or any protonated compounds. Clusters of acrylonitrile (CH_2_=CHCN, from now on denoted as ACN) with water and alcohols are therefore readily formed and could even exist in the interstellar medium and atmospheres of other planets and satellites. To the best of our knowledge, no interstellar neutral hydrogen-bonded cluster has been identified in that environment up to now. Clusters of interstellar molecules with water and methanol, however, appear to be very promising targets of observations, especially in star-forming regions where the warm-up during star formation leads to evaporation of the icy mantles of the interstellar grains that contain considerable amounts of these two compounds.

The most useful spectral range for the unambiguous identification of molecular systems is the microwave and millimeter wave region covering rotational molecular transitions which are considered the molecular fingerprints of species. Because of the higher temperatures prevalent in star-forming regions such clusters are more likely to be detected at higher frequencies probing transitions with higher *J* quantum numbers which are more populated under those circumstances. Nevertheless, extrapolation of existing low-frequency spectra to predict the positions of higher frequency rotational lines and, particularly, their intensity profiles is subject to considerable errors due to the shallow potential energy wells of these clusters, which leads to a more floppy nature than the one of valence-bound molecules and also opens for the existence of several isomers of the cluster.

In the case of weakly bonded clusters such rotational spectra can be studied by supersonic expansion of a monatomic carrier gas containing about few percent of the two moieties of the complex. Rotational studies on clusters of cyano compounds have been undertaken for a multitude of complexes containing HCN (hydrogen cyanide) and a few of those with CH_3_CN (acetonitrile).^4^ In these clusters it has been observed that HCN mainly acts as a proton donor while the electron pair of the nitrogen atom in CH_3_CN often functions as proton acceptor. Published studies of clusters of CH_3_CN include those with H atom donors such as HF,^5,6^ HCl,^7^ HCN,^8^ sand HCCH^9^ but also complexes with electron pair acceptors (Lewis acids) bonding to the lone electron pair at the cyano group, such as BF_3_,^10^ SO_3_,^11^ and, surprisingly, also F_2_^12^ have been reported. In addition, the rotational spectrum of the solvated acetonitrile molecule (CH_3_CN·H_2_O) has been measured.^13^ In all these clusters, the observed rotational spectrum is characteristic for a symmetric top, and for the CH_3_CN·H_2_O cluster, two tunneling components related to the motion of water were observed.

Furthermore, the rotational spectrum of the ACN–water cluster (CH_2_=CHCN·H_2_O, from here on ACN·W) and some of its isotopologues have recently been studied in the 59.6–74.4 GHz range by Calabrese et al.^14^*Ab initio* calculations predicted the existence of three different isomers of the ACN·W cluster in analogy with similar structures of the benzonitrile–water complex.^15^ However, only one of three structural isomers, namely the one in which the water molecule forming 2 HBs (one OH···N bond with the nitrogen atom of ACN and one C–H···O bond with one of the hydrogens of the terminal CH_2_ group of the ACN) has been observed. Thus, the even-membered ring structure of ACN·W resembles the one of benzonitrile–water, but differs from the almost linear structure of CH_3_CN·H_2_O. Furthermore, the calculated dissociation energies are very similar for ACN·W and benzonitrile–water (*D*_e_ = 24.4 and 25.0 kJ mol^–1^, respectively). The spectra showed also the spectroscopic effects of an internal rotation (3:1 intensity ratio of the split lines of the light hydrogen isotope analogue to ortho- and para-hydrogen) of the water moiety. The *ab initio* energy value (MP2/aug-cc-pVTZ level of calculation) reported for the transition state for this motion in ACN·W is 5.54 kJ mol^–1^, which is, again, very similar to the value obtained for benzonitrile–water (5.48 kJ mol^–1^).

It is interesting to compare the ACN·W with the ACN·methanol cluster (CH_2_=CHCN·CH_3_OH, from here on ACN·Met). The geometry of the most stable isomer can be expected to be similar to the one obtained for ACN·W with the hydroxyl group of the methanol acting as a hydrogen donor to the nitrogen atom of the ACN moiety and the oxygen atom of the methanol interacting with one of the hydrogen atoms of ACN. In this context it will be promising to assess how the somewhat lower dipole moment of methanol (1.700 D^16^) compared to water (1.855 D^16^) affects the structure and the bond strength of the cluster. Furthermore, the possibility of the internal rotation of the methyl group adds interest to the ACN·Met system. In previous studies of similar systems (e.g., CH_3_OH·H_2_O^17^), a large discrepancy between the barriers predicted by *ab initio* calculations and the experimentally found ones has been detected, the experimentally found values being considerably smaller than the theoretical ones. In a study by Fraser et al.,^18^ this disagreement was ascribed to the influence of the large amplitude librational movement of the methanol moiety around its *a* inertial axis, disregarding an earlier theory that the lowering of the torsional barrier is due to a modification of the potential due complexation. The ACN·Met cluster can be employed as a test case for this interpretation. Thus, it can serve as a valuable benchmark system for theoretical calculations.

This paper presents a microwave spectroscopy study of the 1:1 ACN·Met cluster in the region between 59.6 and 74.4 GHz. The aim of the study is to measure rotational transitions of the species in the frequency range useful for astronomic detection and to gain detailed insights on the structure and the dynamics of the cluster unraveling the interaction between the two moieties of the complex.

## Theoretical and Experimental Methods

The millimeter-wave (59.6–74.4 GHz) spectrum of ACN·Met was recorded using a Stark modulated free-jet absorption millimeter wave (FJAMMW) spectrometer, the basic design of which has been already described elsewhere.^19,20^ In addition to this design, a new radio frequency source and a ×4 multiplication chain, which has also been described before,^21^ has been currently used.

ACN, CH_3_OH, and CD_3_OD were purchased from Sigma-Aldrich (purity >99%) and used without further purification. The compound and methanol were kept in two separate containers, and both were cooled by a mixture of ice and NaCl (to about −20 °C) while a stream of argon at a pressure of about 200 kPa was passed above them. Under these conditions, the concentrations of ACN and water in the gas mixture were of about 3% and 7%, respectively. The mixture was then expanded from a stagnation pressure of 45 kPa to about 0.5 Pa through a 0.35 mm diameter pinhole nozzle. These settings have been found to be optimal for the formation of the 1:1 complex in our experiments.

Under these conditions, the postexpansion rotational temperature was about 10 K. Electric fields of 750 V cm^–1^ were used to maximize the degree of Stark modulation. Lines separated by more than 300 kHz are resolvable, and the estimated accuracy of the frequency measurements is better than 50 kHz.

Full geometry optimization and evaluation of the Hessian matrix of the monomers and dimers were carried out at the MP2/aug-cc-pVTZ level of calculation. The basis set superposition effect (BSSE) on the obtained geometries was estimated by the counterpoise correction procedure.^22^ Torsional potential energy surfaces were obtained varying the involved dihedral angle whereas all other internal coordinates were freely optimized. All calculations were performed with the GAUSSIAN09 program package.^23^

Complementary information was also achieved visualizing the noncovalent interactions (NCI) with the NCI method^24^ which considers the distribution of both the electron density (ρ), and its gradient (*s*) and its second derivatives matrix (λ_1_, λ_2_, λ_3_). A comprehensive picture can be drawn using different plots of these quantities. According to the color code reported on the graphics, the isosurfaces visible in the NCI plots represent the area for attractive and repulsive interactions.

## Results and Discussion

**Figure 1:**
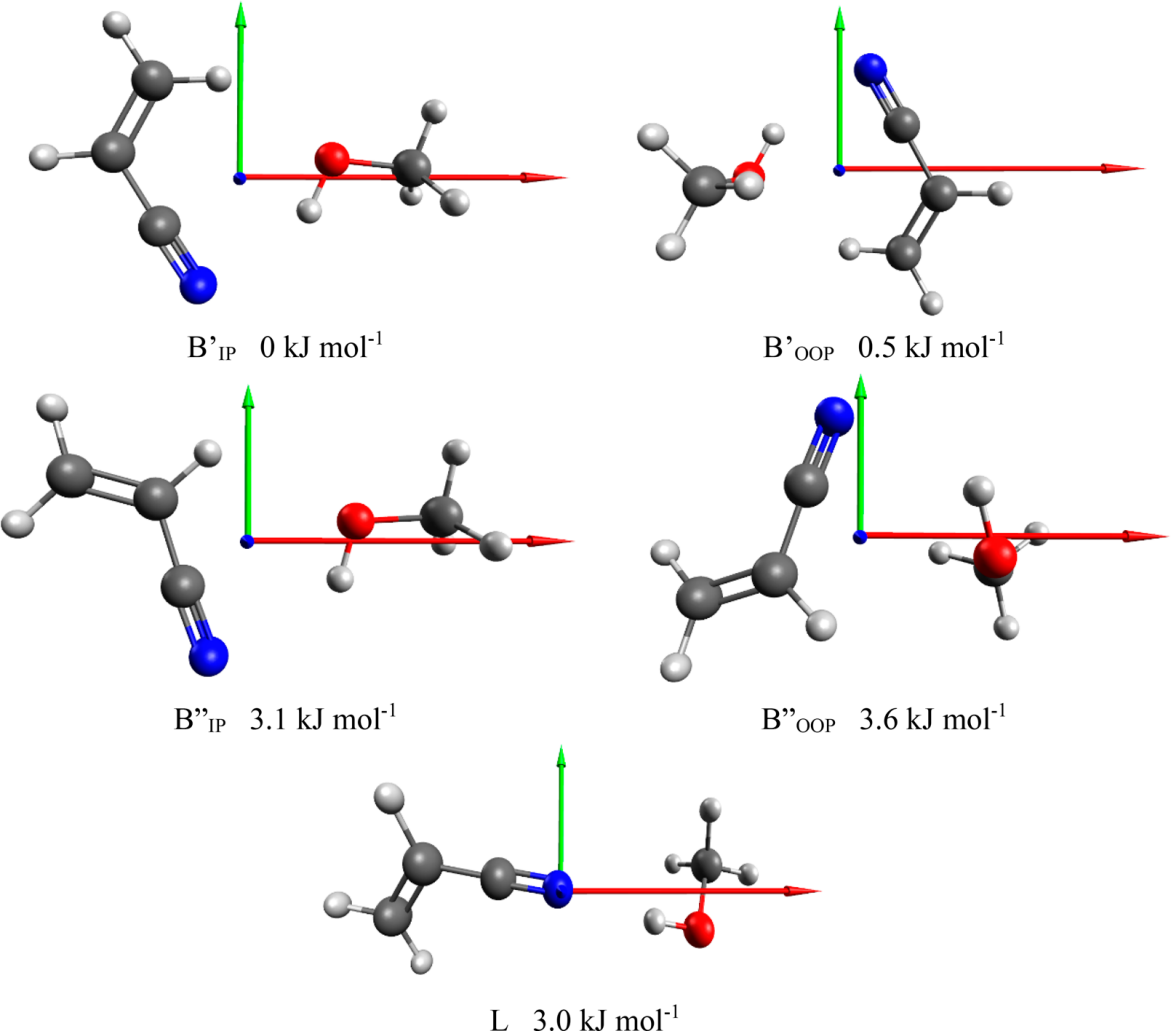
Theoretical molecular structures, principal axis system, and zero-point corrected relative energies for ACN·Met conformers (MP2/aug-cc-pVTZ). The labeling of the conformers refers to the type of interaction (B “bridge” or L “linear”), and to the position between the two moieties: IP “in-plane”, OOP “out-of-plane”. The principal axis are indicated: in red the “a” axis and in green the “b” axis. The “c” axis is perpendicular to the plane. See text for more details.

The conformational space of ACN·Met was explored running several *ab initio* optimization procedures, each of them starting from a different initial geometry. The choice of the starting geometries were informed by previous studies such the already mentioned one on ACN·W.^14^ In that case two planar forms, in which the water acts as both proton donor to the CN group and proton acceptor from a C–H bond, and a conformation characterized by an almost linear H···N hydrogen with the unbound hydrogen atom lying out of the heavy atom plane, were identified. In the ACN·Met case five structural minima were identified and are shown in Figure 1. In all conformations but linear (L), the methanol hydroxylic hydrogen is directed toward the cyano group while the oxygen is engaged in a secondary interaction with one of the ethylenic hydrogen atoms, forming a bridged structure (B). In two cases, the methanol moiety is on the same side with respect to the double bond (B′ conformers) while in the other two it is situated on the opposite side (B″ conformers). The two subgroups of conformers are separated by an energy gap that spans about 3–4 kJ mol^–1^ depending on taking or not into account the zero point energy. Within each of the two subgroups the lowest energy conformer is the one in which the carbon of the methyl group lies in the ACN plane (conformers B′_IP_ and B″_IP_) while the highest energy sees the methyl group perpendicular to this plane (conformers B′_OOP_ and B″_OOP_). The L conformation differs from all the others since the methanol shows an almost linear O–H···CN bond and no other interaction with the ACN. All calculated spectroscopic parameters (rotational and quartic centrifugal distortion constants), electric dipole moments components, relative and binding energies, and their corresponding zero point corrected quantities are reported in Table 1. Due to the presence of the nitrogen atom, it is expected that the rotational spectrum will show a hyperfine structure caused by the coupling of the nitrogen nucleus quadrupole moment to the overall molecular rotation. For this reason, the nuclear quadrupole coupling constants have also been calculated and are reported in the same table.

**Table 1:** Relative Energies, Dissociation Energies, and Spectroscopic Parameters (Rotational Constants, Centrifugal Distortion Constants, Dipole Moment Components, and Nuclear Hyperfine Coupling Constants) for Different Conformers of ACN·Met (MP2/aug-cc-pVTZ)

	B′_IP_	B′_OOP_	L	B″_IP_	B″_OOP_
*A*/MHz	5051	4262	16502	6079	4908
*B*/MHz	1670	2177	826	1395	1768
*C*/MHz	1265	1609	813	1143	1434
*D*_*J*_/kHz	1.04	4.33	1.15	0.96	4.86
*D*_*JK*_/kHz	5.61	–1.48	–107.54	0.01	–21.92
*D*_*K*_/kHz	2.93	4.54	1.41	81.75	86.65
*d*_1_/kHz	–0.33	–1.20	0.39	–0.29	–1.34
*d*_2_/kHz	–0.08	–0.09	–0.14	–0.04	–0.00
μ_*a*_/D	–0.91	1.80	–5.74	–0.44	–1.57
μ_*b*_/D	2.11	–2.41	1.61	2.36	–2.49
μ_*c*_/D	0	1.43	–0.79	0	–0.93
μ_*tot*_/D	2.30	3.33	6.01	2.40	3.08
χ_*aa*_/MHz	0.26	0.59	–3.55	1.36	1.30
χ_*bb*_/MHz	–2.07	–2.38	1.72	–3.20	–3.09
χ_*cc*_/MHz	1.80	1.80	1.83	1.85	1.80
(*χ*_*bb*_ – *χ*_*cc*_)/MHz	–3.87	–4.18	–0.11	–5.05	–4.89
*ΔE*/kJ mol^–1^	0^a^	0.22	3.48	3.91	4.38
*ΔE*_0_/kJ mol^–1^	0^b^	0.48	3.03	3.10	3.63
*D*_e_/kJ mol^–1^	26.3	26.05	22.80	22.4	21.89
*D*_0_/kJ mol^–1^	22.01	21.53	18.99	18.91	18.38

a Absolute energy value: −286.033014 hartree.

b Absolute energy value: −285.929159 hartree.

Based on the theoretical data, the prediction of the rotational spectra of all conformers were obtained in the frequency range covered by the FJAMMW spectrometer. The most intense components of the spectrum were expected to be *R*-branch μ_b_-type transitions lines of the most stable conformations B′_IP_ and B′_OOP_. In the case of the out-of-plane conformer, the presence of a plane of symmetry (coincident with the ACN plane) creates the possibility of having two equivalent conformations related to the position of the methyl group on different sides of the plane. Thus, the spectrum originated from the second conformer is expected to be more intense due to this double degeneracy caused by symmetry. A series of such lines was indeed observed while no μ_a_- or μ_c_-type lines were detected. The first ones are because of the high rotational quantum numbers of the energy levels involved which would be depopulated in the free jet expansion, and the second ones are in agreement with the null value of the expected dipole moment for *C*_*s*_ molecular symmetry. The measured lines have rotational quantum number *J* ranging from *J* = 7 up to *J* = 18 and *K*_a_ ranging from *K*_a_ = 4 to *K*_a_ = 7. The lower *J* transitions were observed in a single scan while tens of scans were accumulated in order to obtain more accurate data and to extend the measurements also to weaker μ_b_-type rotational transitions originating from higher rotational quantum numbers. All rotational transitions were split by a tunneling effect due to the hindered internal rotation of the methyl group in an A nondegenerate level and an E doubly degenerate one. A second set of experiments using CD_3_OD in place of methanol has been carried out in the same conditions and a similar set of transitions was observed in the recorded free jet spectrum.

The analysis of the spectra was performed using the XIAM program.^25^ This code is based on the combined axis method (CAM),^26^ and directly supplies the *V*_3_ barrier to internal rotation, the angles between the internal rotation axis and the principal axes, and the moment of inertia of the internal top. As regards the spectroscopic constants, a set common to both the A- and E-states is provided, corresponding to the values for the infinite barrier limit. The rotational Hamiltonian includes the *S*-reduced Watson’s semirigid Hamiltonian in the *I*^r^ representation^27^ and includes the nuclear quadrupole coupling Hamiltonian. All transitions’ frequencies are reported in the Supporting Information, while the spectroscopic parameters directly deduced from the fitting procedures are reported in Table 2. The standard deviations (0.09 and 0.06 MHz for the parent and the deuterated species, respectively) are only slightly above the estimated experimental uncertainty (0.05 MHz). This result is surely acceptable, especially considering that due to the hyperfine coupling some lines are blended. In the fitting procedure the nuclear quadrupole coupling constant χ_aa_ was fixed to the *ab initio* value. Even though the fitting results were insensitive to this constant, the standard deviation was slightly better than that obtained fixing the constant to zero.

**Table 2:** Spectroscopic Constants (Rotational Constants, Centrifugal Distortion Constants, Nuclear Quadrupole Coupling Constants, and Internal Rotation Constants) of the Observed ACN·Met Conformation

	ACN–CH_3_OH	ACN-CD_3_OD
*A*/MHz	5008.05(5)	4840.21(2)
*B*/MHz	1628.89(1)	1457.690(5)
*C*/MHz	1239.26(1)	1136.959(8)
*D*_*J*_/kHz	1.24(3)	1.032(6)
*D*_*JK*_/kHz	8.4(3)	5.64(3)
*D*_*K*_/kHz	3.0(4)	2.94(8)
*d*_*1*_/kHz	–0.387(9)	–0.262(3)
*d*_*2*_/kHz	–0.080(7)	–0.0598(9)
*H*_*KJ*_/kHz	0.014(5)	[0]^b^
*χ*_*aa*_/MHz	[0.26]^b^	[0.26]^b^
(*χ*_*bb*_ – *χ*_*cc*_)/MHz	–4.2(3)	–4.6(1)
*V*_3_/kJ mol^–1^	2.654(9)	2.61(6)
*V*_3_/cm^–1^	221.9(7)	218(5)
*F*_0_/GHz	157.9(4)	77(1)
δ/deg	3.24(3)	2.86(6)
σ^c^/MHz	0.09	0.06
*N*^d^	76	85
Δ_c_^e^/uÅ	–3.366	–6.611

a Error in parentheses in units of the last digit.

b Fixed to the indicated value.

c Root-mean-square deviation of the fit.

d Number of lines in the fit.

e Inertia defect Δ_c_= *I*_c_ – *I*_a_ – *I*_b_, conversion factor 505379.05 MHz uÅ^2^.

In Table 2 the inertia defect for the two species are also reported. Their values are consistent with an overall planar structure of the complex where only the methyl hydrogen atoms are out of plane. In fact, they are very similar to the moment of inertia of the methyl top calculated *ab initio* for this complex (3.29 uÅ^2^ for ACN–CH_3_OH and 6.53 uÅ^2^ ACN–CD_3_OD respectively).

**Figure 2:**
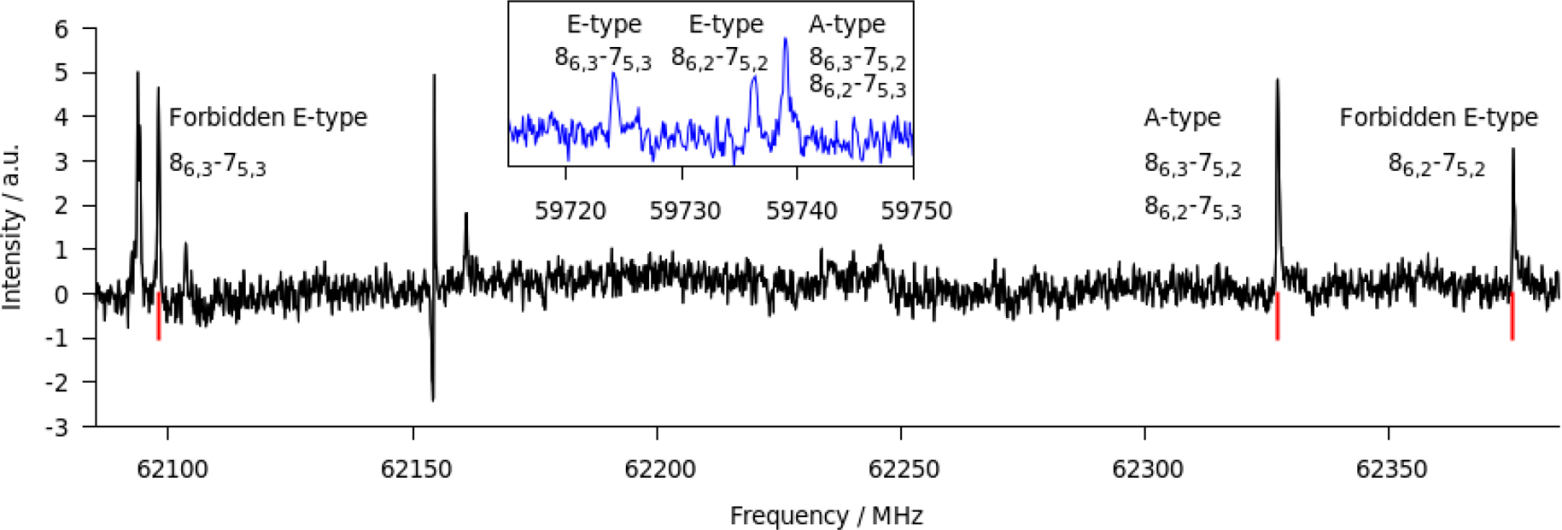
Examples of the internal rotation patterns for rotational transitions originating from ACN·CH_3_OH (black spectrum) and ACN·CD_3_OD (blue spectrum). The rotational transitions shown in the blue spectrum are the same as those labeled in the black one.

Examples of the measured rotational transitions showing the internal rotation pattern for the normal and deuterated species of ACN·Met are shown in Figure 2. It can be seen how the internal rotation splitting reduces upon deuteration of the methyl group due to the lower value of the reduced mass of the motion. The unlabeled lines present in the picture can be attributed to methanol and its dimer or to unknown species. Attempts to assign the unknown lines to other conformations of ACN·Met have been performed but were unsuccessful.

**Figure 3:**
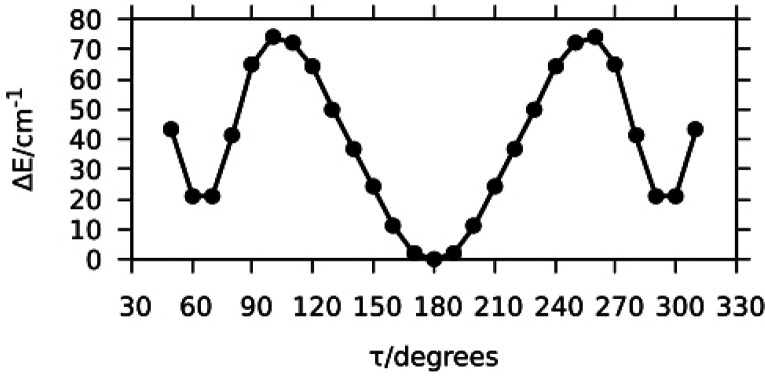
*Ab initio* potential energy surface for the torsion of the methanol’s methyl group around the OH bond for the B′ conformations. This motion interconverts the in-plane conformer (absolute minimum, B′_IP_) with the out-of-plane ones (relative equivalent minima, B′_OOP_).

The comparison between the experimentally determined constants and those from *ab initio* calculations allows the identification of the observed species with conformer B′_IP_. This assignment is based on the comparison of different findings: the rotational constants, the centrifugal distortion constants, the types of transitions observed which are linked to the dipole moment components, the nuclear quadrupole coupling constants, and the angles formed by the rotating methyl group with the molecular frame. Such angles are used as initial inputs for the fitting procedure (*ab initio* values) but can also be fitted to reproduce the experimental data. The rotational transitions of the B′_OOP_ conformer were unsuccessfully searched for in the spectrum notwithstanding their predicted higher intensity. This can be attributed to a relaxation of the population of this conformation onto that of the global minimum during the adiabatic expansion. This is justified by the low barrier (around 50 cm^–1^) to interconversion between the two conformations, which has been calculated *ab initio* and is depicted in Figure 3. The potential energy surface was calculated varying the dihedral angle on a regular grid with a step of 10°.

**Figure 4:**
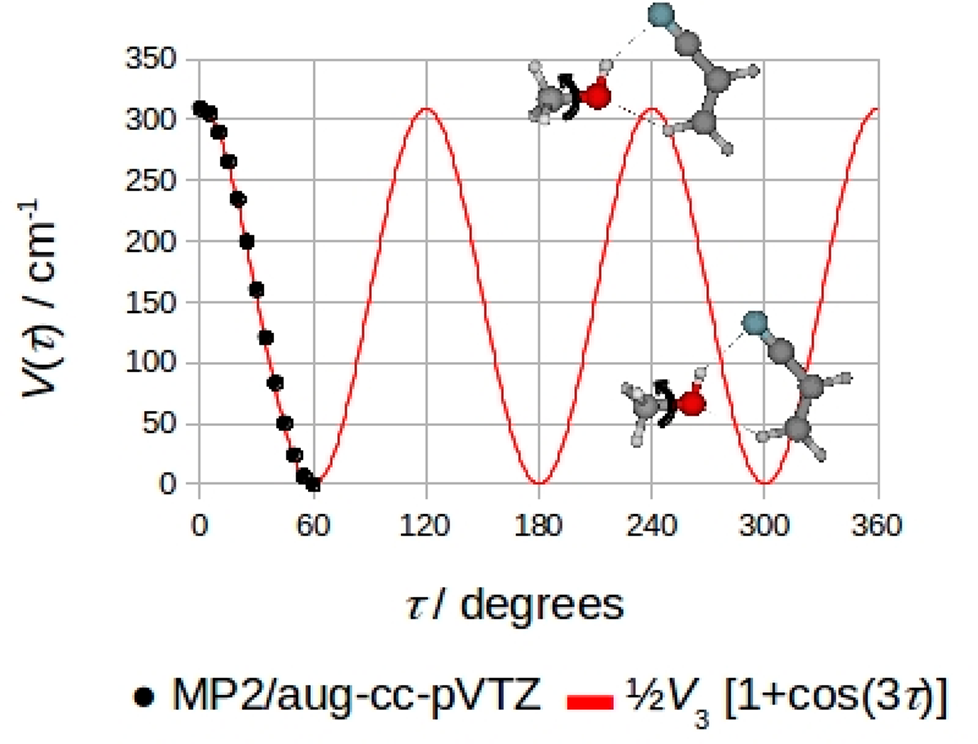
*Ab initio* methyl internal rotational potential energy surface for ACN·Met B′_IP_ molecular complex.

After the assignment to the correct structure, the potential energy surface for the torsion of the methanol’s methyl group around the OC bond depicted in Figure 4 was calculated varying the τ = HC–OH dihedral angle on a regular grid with Δτ = 5°. The computed data, represented as black bullets, are well reproduced by the simple 3-fold function: *V*(τ) = ^1^/_2_*V*_3_[1 + cos(3τ)], which is shown with a red line in Figure 4. The value of the maximum (311 cm^–1^) represents the theoretical barrier hindering the methyl group internal rotation in the complex, and it can be noted that it is quite a bit larger (about 41%) than the experimental values obtained for the normal species, *V*_3_ = 221.9(7) cm^–1^, and for ACN–CD_3_OD, *V*_3_ = 218(5) cm^–1^ (see Table 2). It is possible to attribute these differences to the accuracy of the theoretical method and this can be tested by calculating the barrier to internal rotation for free methanol (348 cm^–1^) and comparing it to the experimental barrier (373.594(7) cm^–1^).^28^ The calculated barrier for free methanol is indeed lower than the experimental one but only by about 7%, which is quite far from the 41% decrease observed in our data.

The analysis of the literature data on the subject shows that this phenomenon has largely been observed. In complexes of methanol where one HB is formed, namely the complexes with H_2_O (*V*_3_ = 60 cm^–1^),^17^ Ar (*V*_3_ = 68.5 cm^–1^),^29^ HCl (*V*_3_ = 74 cm^–1^),^30^ SO_2_ (*V*_3_ = 128.6 cm^–1^),^31^ phenol (*V*_3_ = 170),^32^ and CO_2_ (*V*_3_ = 174.8 cm^–1^)^33^ a lowering from 50% to 84% of the experimental barrier with respect to the one in the methanol monomer has been observed. In the dimer of methanol,^34^ two barriers were determined for the methanol acting as the proton acceptor (*V*_3_ = 120 cm^–1^) or proton donor (*V*_3_ = 183 cm^–1^) which are lower than that of free methanol by 68% and 51% respectively. When two HBs are formed such as in the complexes of methanol with aniline (*V*_3_ = 215 cm^–1^),^35^ formamide (*V*_3_ = 231 cm^–1^),^36^ and formaldehyde (*V*_3_ = 240.5 cm^–1^)^33^ the lowering is between 36% and 42%, similar to the case of ACN (41%) studied in this work. A systematic study of many of these systems was performed by Fraser et al.^18^ in a note where they show that the decreasing of the barrier is only apparent and it is an artifact related to the large amplitude librational motion of the methanol moiety about the *a* inertial axis, which in fact changes the effective reduced mass of the methyl group with respect to the frame.

To test this idea, we can consider our data on the complex of ACN with CD_3_OD which was obtained using CD_3_OD instead of CH_3_OH to form the complex. We assigned a set of lines with quantum numbers similar to those of the normal species and determined all spectroscopic constants including the moment of inertia (*F*_0_) and the barrier to internal rotation (*V*_3_) of the deuterated methyl group. As can be seen from the inspection of Table 2, the value for the barrier (218(5) cm^–1^) is very similar to that of the normal species (221.9(7) cm^–1^) and the moment of inertia of the rotating methyl group (77(1) GHz) is very similar to the initial theoretical value calculated from the *ab initio* structure (77.4 GHz). We are aware that some water can be present in the sample and in the system, thus we cannot exclude that some isotopic exchange with the deuterated hydroxylic group could take place. For this reason, we must account that the assigned spectrum could belong to ACN·CD_3_OH instead of ACN·CD_3_OD; indeed, the difference between the rotational constants of the −OH and −OD species is calculated to be about 0.3%, which is smaller than the difference between the calculated and observed constants.

This uncertainty regarding the observed deuterated species is not significant regarding the reduction in the observed barrier relative to theory since the *F*_0_ value determined from the internal rotation splittings is very close to the one calculated theoretically for the CD_3_ internal rotor and the *V*_3_ barrier to that of the undeuterated species. This fact led us to exclude that, in the particular case of ACN**·**Met, the decrease of the methanol internal rotation barrier is related to a librational motion of the whole methanol moiety or the methyl group, because in that case we would see a consistent change in the determined internal rotation parameters upon deuteration of the whole methyl group. Nevertheless, from our data, a motion of the hydroxyl group cannot be excluded.

**Figure 5:**
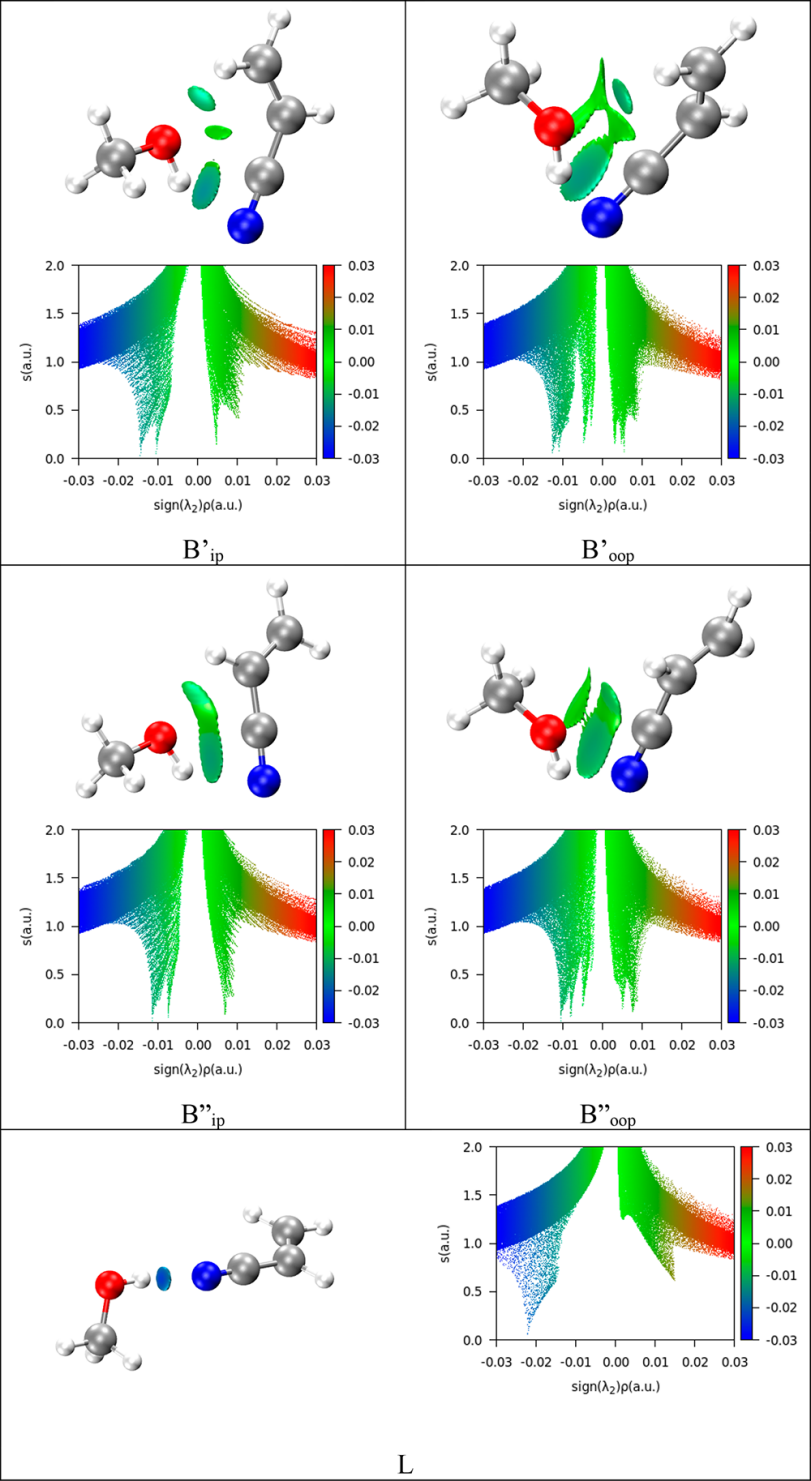
NCI plots from the *ab initio* outputs for all conformers of ACN·Met. Upper diagrams: blue and green colors identify the presence of strong and weak attractive interactions, respectively. Red color indicates repulsive interaction. Lower diagrams value of the electron density gradient (*s*) vs the electron density (ρ) multiplied by the sign of its second derivative λ_2_. Positive values indicate repulsive interactions, and negative values attractive ones.

The observed species (B′_IP_), which is also the global minimum among the possible conformations, is stabilized by the formation of a ring like structure between the methanol and ACN driven by noncovalent interaction between the two moieties. A visualization of the noncovalent interactions was achieved with the NCI method.^37^ A comprehensive picture can be drawn using different plots of these quantities (see Figure 5). According to the color code reported on the graphics, the isosurfaces visible in the NCI plots represent the area for attractive (blue) and repulsive (red) interactions. From Figure 5, we can infer that in the minimum energy configurations of the ACN**·**Met complex there are two attractive interactions (points where *s* = 0 and λ_2_ is negative): an attractive O–H···CN interaction (that with the largest value of ρ) and a weaker C–H···O one. The comparison between the lowest energy member of the B″ family (in plane) indicates that the HBs in the global minimum are less diffuse and slightly stronger in B′ than in B″. The interactions are similar to those observed in other hydrogen-bonded complexes with cyano-systems such as benzonitrile–water and ACN·W. In all cases, a cyclic form stabilized by two intermolecular interactions is also formed: a HB from water or methanol toward the cyano group and a secondary CH···O HB to the oxygen atom of water. The OH···N and CH···O distances can be compared to in these systems. They are 2.36 and 2.22 Å in ACN·Met (MP2/aug-cc-pVTZ values), 2.257(1) and 2.484(1) Å in benzonitrile–water^15^ and 2.331(3) and 2.508(4) Å in ACN·W.^14^

Other complexes with methanol, e.g., formamide–methanol^36^ and formaldehyde–methanol,^33^ have been characterized by rotational spectroscopy and the structure of the global minimum is stabilized by similar cyclic structures. In formamide–methanol there is a HB from the water hydrogen to the oxygen atom of formamide and one from the N–H group of formamide to the water oxygen. The distances are quite short: 2.01(1) and 1.97(1) Å, respectively. In formaldehyde–methanol, the HBs are from the water to the oxygen of formaldehyde (2.097(6) Å) and from a CH group of the second moiety to the water oxygen. The shorter distances in formamide–methanol seem to imply a larger binding energy which is indeed confirmed by the *ab initio* (MP2/aug-cc-pVTZ) value of 44.5 kJ mol^–1^ for this system with respect to the corresponding values for ACN·Met (26.3 kJ mol^–1^) and formaldehyde·Met (24.1 kJ mol^–1^) which are very similar to one another.

## Conclusions

We report on the investigation of the rotational spectrum of the 1:1 adduct formed between ACN and methanol and its fully deuterated form (CD_3_OD). The rotational spectra have been recorded and analyzed in the millimeter wave region (59.6–74.4 GHz) and could be used to identify the complex in astronomical observations. The determined rotational constants are coherent with a theoretical determined cyclic structure (MP2/aug-cc-pVTZ) stabilized by a primary OH···N HB between water and the cyano group and a secondary one between the terminal CH group and the water oxygen which were also characterized and visualized using NCI plots.

Splittings of the rotational lines due to the hindered internal rotation of the methyl group were observed and the global analysis of the spectrum leads to the determination of a *V*_3_ value of 221.9(7) and 218(5) cm^–1^ for the complexes of CH_3_OH and CD_3_OD, respectively. These values are about 40% lower than that experimentally determined for free methanol. The correspondence of the reduced mass of the internal rotation motion to the one calculated from the structure, for both the normal and the deuterated species, led us to exclude that the lowering of the barrier could be ascribed to a librational motion of the whole methyl group.

## Supporting Information Available

Complete reference (23), tables of experimental frequencies for the acrylonitrile-methanol complexes (ACN-CH_3_OH, Table S1 and ACN-CD_3_OD, Table S2) are supplied as Supporting Information (PDF)
