# Supplementary material for: Structure, Dynamics, and Accurate Laboratory Rotational Frequencies of the Acrylonitrile–Methanol Complex
Source: J Phys Chem A. 2020 Apr 11;124(18):3601–8. doi: 10.1021/acs.jpca.0c01334 (PMC8007102; doi:10.1021/acs.jpca.0c01334)
Supplement: Supplementary file 1 — jp0c01334_si_001.pdf [file jp0c01334_si_001.pdf]

Structure, Dynamics and Accurate Laboratory Rotational Frequencies of the Acrylonitrile-Methanol Complex

Camilla Calabrese,[1,2,3] Assimo Maris,[1] Annalisa Vigorito,[1] Sergio Mariotti,[4] Pantea Fathi,[5] Wolf D. Geppert[5,*] and Sonia Melandri[1,*]

[1]Dipartimento di Chimica "G. Ciamician" dell'Università, via Selmi 2, I-40126 Bologna, Italy

[2] Current address: Departamento Química Física, Facultad de Ciencia y Tecnología Universidad del País Vasco (UPV/EHU), Apartado 644, E-48080 Bilbao, Spain.

[3] Current address: Biofisika Institute, (CSIC, UPV/EHU) University of the Basque Country (UPV/EHU), Barrio Sarriena, S/N, 48940, Leioa (Spain)

[4] INAF - Osservatorio di Radioastronomia, via P. Gobetti, 101, I-40129 Bologna, Italy

[5] Department of Physics, Stockholm University, Albanova University Center, SE-106 91 Stockholm, Sweden

Supporting Information

Complete reference 23

Table S1. Experimental transition frequencies of ACN·CH_3OH

Table S2. Experimental transition frequencies of ACN·CD_3OD

Reference 23

Gaussian 09, Revision D.01, M. J. Frisch, G. W. Trucks, H. B. Schlegel, G. E. Scuseria, M. A. Robb, J. R. Cheeseman, G. Scalmani, V. Barone, G. A. Petersson, H. Nakatsuji, X. Li, M. Caricato, A. Marenich, J. Bloino, B. G. Janesko, R. Gomperts, B. Mennucci, H. P. Hratchian, J. V. Ortiz, A. F. Izmaylov, J. L. Sonnenberg, D. Williams-Young, F. Ding, F. Lipparini, F. Egidi, J. Goings, B. Peng, A. Petrone, T. Henderson, D. Ranasinghe, V. G. Zakrzewski, J. Gao, N. Rega, G. Zheng, W. Liang, M. Hada, M. Ehara, K. Toyota, R. Fukuda, J. Hasegawa, M. Ishida, T. Nakajima, Y. Honda, O. Kitao, H. Nakai, T. Vreven, K. Throssell, J. A. Montgomery, Jr., J. E. Peralta, F. Ogliaro, M. Bearpark, J. J. Heyd, E. Brothers, K. N. Kudin, V. N. Staroverov, T. Keith, R. Kobayashi, J. Normand, K. Raghavachari, A. Rendell, J. C. Burant, S. S. Iyengar, J. Tomasi, M. Cossi, J. M. Millam, M. Klene, C. Adamo, R. Cammi, J. W. Ochterski, R. L. Martin, K. Morokuma, O. Farkas, J. B. Foresman, and D. J. Fox, Gaussian, Inc., Wallingford CT, 2016.

Table S1. Experimental transition frequencies of ACN·CH_3OH

								A lines	
J'	K_a'	K_c'	J''	K_a''	K_c''	F′	F″	ν (MHz)	ν_{obs}-$\nu_{cal.}$ (MHz)
7	7	1	6	6	0			66600.36	0.01
7	7	0	6	6	1			66600.36	0.01
8	7	2	7	6	1			69475.41	-0.08
8	7	1	7	6	2			69475.41	-0.08
9	7	3	8	6	2			72348.57	0.04
9	7	2	8	6	3			72348.57	0.03
8	6	3	7	5	2			62327.29	0.13
8	6	2	7	5	3			62327.29	-0.05
9	6	4	8	5	3			65194.74	0.19
9	6	3	8	5	4			65195.23	-0.10
10	6	5	9	5	4			68053.86	0.13
10	6	4	9	5	5			68056.62	0.14
11	6	6	10	5	5			70900.36	-0.13
11	6	5	10	5	6			70908.73	0.00
12	6	7	11	5	6			73728.77	0.02
12	6	6	11	5	7			73750.72	0.06
10	5	6	9	4	5			60800.23	0.02
10	5	5	9	4	6			60892.69	-0.07
11	5	7	10	4	6			63544.56	0.09
11	5	6	10	4	7			63758.46	-0.06
12	5	8	11	4	7			66195.40	-0.03
12	5	7	11	4	8			66647.01	0.00
13	5	9	12	4	8			68704.92	0.07
13	5	8	12	4	9			69586.30	0.08
14	5	10	13	4	9	30	28	71011.77	-0.70
14	5	9	13	4	10			72618.79	-0.01
15	5	11	14	4	10	32	30	73053.18	0.02
15	5	11	14	4	10	30	28	73052.74	-0.11
15	5	11	14	4	10	28	26	73053.18	0.00

12	4	8	11	3	9			61080.04	-0.06
13	4	9	12	3	10	28	26	65016.43	-0.06
13	4	9	12	3	10	26	24	65016.74	-0.09
13	4	9	12	3	10	24	22	65016.43	-0.04
14	4	10	13	3	11	30	28	69435.60	-0.04
14	4	10	13	3	11	28	26	69436.09	-0.01
14	4	10	13	3	11	26	24	69435.60	0.14
15	4	12	14	3	11	32	30	60281.27	-0.07
15	4	12	14	3	11	30	28	60280.65	-0.11
15	4	12	14	3	11	28	26	60281.27	-0.11
16	4	13	15	3	12	34	32	61057.83	0.01
16	4	13	15	3	12	32	30	61057.24	0.00
16	4	13	15	3	12	30	28	61057.83	-0.03
18	4	15	17	3	14	38	36	62364.65	0.01
18	4	15	17	3	14	36	34	62364.22	0.10
18	4	15	17	3	14	34	32	62364.65	-0.02
								E lines	
J′	K_a′	K_c′	*J*′′	K_a′′	K_c′′	F′	F″	ν (MHz)	ν_{obs}-$\nu_{cal.}$ (MHz)
7	7	1	6	6	1			66359.65	0.14
7	7	0	6	6	0			66629.17	-0.06
8	7	2	7	6	2			69234.73	0.10
9	7	3	8	6	3			72107.54	-0.08
9	7	2	8	6	2			72377.50	0.00
8	6	3	7	5	3			62097.89	-0.08
8	6	2	7	5	2			62375.61	0.00
9	6	4	8	5	4			64965.40	-0.05
9	6	3	8	5	3			65243.30	-0.17
10	6	5	9	5	5			67825.18	-0.08
10	6	4	9	5	4			68103.80	-0.12
11	6	6	10	5	6			70674.22	0.00
11	6	5	10	5	5			70953.93	0.07
12	6	7	11	5	7			73508.47	-0.07
12	6	6	11	5	6			73789.54	0.01
10	5	6	9	4	6			60628.97	-0.03
10	5	5	9	4	5			60914.40	0.01
11	5	7	10	4	7			63438.66	-0.08
11	5	6	10	4	6			63714.24	-0.03
12	5	8	11	4	8			66232.39	0.15
12	5	7	11	4	7			66459.55	-0.05
13	5	9	12	4	9			69038.01	-0.06
13	5	8	12	4	8			69101.67	0.15
14	5	10	13	4	10			71912.75	0.02
14	5	9	13	4	9			71566.05	0.12
15	5	11	14	4	11	32	30	73794.81	0.03
15	5	11	14	4	11	30	28	73794.43	-0.10

15	5	11	14	4	11	28	26	73794.81	0.01
12	4	8	11	3	8			59998.84	0.04
13	4	10	12	3	10			63410.69	0.21
13	4	9	12	3	9			59612.00	0.22

Table S2. Experimental transition frequencies of ACN·CD_3OD

								A lines	
J′	K_a′	K_c′	*J*′′	K_a′′	K_c′′	F′	F″	ν (MHz)	ν_{obs}-$\nu_{cal.}$ (MHz)
8	8	0	7	7	1			73893.90	-0.01
8	8	1	7	7	0			73893.90	-0.01
7	7	0	6	6	1			64218.66	-0.08
7	7	1	6	6	0			64218.66	-0.08
8	7	1	7	6	2			66817.94	0.06
8	7	2	7	6	1			66817.94	0.06
9	7	2	8	6	3			69415.59	0.05
9	7	3	8	6	2			69415.59	0.05
10	7	3	9	6	4			72010.79	-0.03
10	7	4	9	6	3			72010.79	-0.01
8	6	2	7	5	3			59738.91	-0.03
8	6	3	7	5	2			59738.91	0.04
9	6	3	8	5	4			62333.07	-0.08
9	6	4	8	5	3			62333.07	0.23
10	6	4	9	5	5			64922.41	-0.06
10	6	5	9	5	4			64921.41	0.01
11	6	5	10	5	6			67505.27	0.02
11	6	6	10	5	5			67501.98	-0.04
12	6	6	11	5	7			70079.90	-0.03
12	6	7	11	5	6			70071.30	-0.03
13	6	7	12	5	8			72645.40	0.00
13	6	8	12	5	7			72624.53	-0.06
11	5	6	10	4	7			60405.31	-0.04
11	5	7	10	4	6			60303.79	-0.08
12	5	7	11	4	8			62991.44	-0.07
12	5	8	11	4	7			62775.86	-0.02
13	5	8	12	4	9			65595.15	0.01
13	5	9	12	4	8			65170.04	0.11
14	5	10	13	4	9			67450.29	0.04
14	5	9	13	4	10			68236.50	0.00
15	5	11	14	4	10			69572.58	0.03
15	5	10	14	4	11			70945.59	0.06
13	4	9	12	3	10	24	22	59979.78	0.14
13	4	9	12	3	10	26	24	59979.78	-0.12
13	4	9	12	3	10	28	26	59979.78	0.12
14	4	10	13	3	11	26	24	63464.61	-0.04
14	4	10	13	3	11	30	28	63464.61	-0.07
14	4	10	13	3	11	28	26	63464.97	-0.05
15	4	11	14	3	12	28	26	67326.78	0.01
15	4	11	14	3	12	32	30	67326.78	-0.02
15	4	11	14	3	12	30	28	67327.28	0.01

16	4	12	15	3	13	30	28	71643.06	0.01
16	4	12	15	3	13	34	32	71643.06	-0.03
16	4	12	15	3	13	32	30	71643.71	0.02
17	4	14	16	3	13	32	30	59976.06	-0.42
17	4	14	16	3	13	36	34	59976.06	-0.38
17	4	14	16	3	13	34	32	59976.06	0.25
18	4	15	17	3	14	36	34	60623.98	0.63
18	4	15	17	3	14	34	32	60623.98	-0.02
18	4	15	17	3	14	38	36	60623.98	0.02
13	3	10	12	2	11	24	22	60996.28	0.07
13	3	10	12	2	11	28	26	60996.28	-0.01
13	3	10	12	2	11	26	24	60997.44	0.05
14	3	11	13	2	12	26	24	66714.79	-0.03
14	3	11	13	2	12	30	28	66714.79	-0.12
14	3	11	13	2	12	28	26	66716.10	-0.02
15	3	12	14	2	13	28	26	72917.44	0.07
15	3	12	14	2	13	32	30	72917.44	-0.01
15	3	12	14	2	13	30	28	72916.20	-2.55
15	4	11	14	3	12	30	28	67327.28	0.01
16	4	12	15	3	13	30	28	71643.06	0.01
16	4	12	15	3	13	34	32	71643.06	-0.03
16	4	12	15	3	13	32	30	71643.71	0.02
17	4	14	16	3	13	32	30	59976.06	-0.42
17	4	14	16	3	13	36	34	59976.06	-0.38
17	4	14	16	3	13	34	32	59976.06	0.25
18	4	15	17	3	14	36	34	60623.98	0.63
18	4	15	17	3	14	34	32	60623.98	-0.02
18	4	15	17	3	14	38	36	60623.98	0.02
13	3	10	12	2	11	24	22	60996.28	0.07
13	3	10	12	2	11	28	26	60996.28	-0.01
13	3	10	12	2	11	26	24	60997.44	0.05
14	3	11	13	2	12	26	24	66714.79	-0.03
14	3	11	13	2	12	30	28	66714.79	-0.12
14	3	11	13	2	12	28	26	66716.10	-0.02
15	3	12	14	2	13	28	26	72917.44	0.07
15	3	12	14	2	13	32	30	72917.44	-0.01
15	3	12	14	2	13	30	28	72916.20	-2.55
								E lines	
J'	K_a'	K_c'	*J''*	K_a''	K_c''	F'	F''	ν (MHz)	ν_{obs}-$\nu_{cal.}$ (MHz)
8	8	0	7	7	0			73887.33	0.01
8	8	1	7	7	0			73878.13	-0.02
7	7	0	6	6	0			64213.94	-0.08
7	7	1	6	6	1			64203.24	-0.05
8	7	1	7	6	1			66813.18	0.02

8	7	2	7	6	2			66802.45	0.01
9	7	2	8	6	2			69410.88	0.05
9	7	3	8	6	3			69400.16	0.05
10	7	3	9	6	4			72006.1	0.01
10	7	4	9	6	4			71995.4	0.02
8	6	2	7	5	2			59736.18	0.04
8	6	3	7	5	3			59724.04	0.01
9	6	3	8	5	3			62330.30	0.07
9	6	4	8	5	4			62318.16	0.04
10	6	4	9	5	4			64919.14	-0.03
10	6	5	9	5	5			64907.03	-0.02
11	6	5	10	5	5			67500.84	-0.02
12	6	6	11	5	6			70072.69	0.01
12	6	7	11	5	7			70061.03	0.10
13	6	7	12	5	7			72631.04	0.00
13	6	8	12	5	8			72621.20	-0.09
11	5	6	10	4	6			60328.45	-0.07
11	5	7	10	4	7			60365.79	-0.01
12	5	7	11	4	7			62811.40	0.05
12	5	8	11	4	8			62941.13	0.02
13	5	8	12	4	8			65223.55	-0.06
13	5	9	12	4	9			65526.40	-0.09
